# Acute cor pulmonale in Covid-19 related acute respiratory distress syndrome

**DOI:** 10.1186/s13054-021-03756-6

**Published:** 2021-09-25

**Authors:** Pedro Cavaleiro, Paul Masi, François Bagate, Thomas d’Humières, Armand Mekontso Dessap

**Affiliations:** 1 AP-HP (Assistance Publique-Hôpitaux de Paris), Hôpitaux universitaires Henri Mondor, DMU Médecine, Service de Médecine Intensive Réanimation, 94010 Créteil, France; 2 UPEC (Université Paris Est Créteil), Faculté de Santé de Créteil, IMRB, GRC CARMAS, 94010 Créteil, France; 3 AP-HP, Hôpitaux universitaires Henri Mondor, Service de Physiologie, 94010 Créteil, France; 4 INSERM, Unité U955, 94010 Créteil, France

Right ventricle (RV) dysfunction is a frequent complication of acute respiratory distress syndrome (ARDS). Its more severe presentation, acute *cor pulmonale* (ACP), is defined at echocardiography as a dilated RV (end-diastolic RV/left ventricle area ratio > 0.6) associated with the presence of septal dyskinesia. The prevalence of ACP in non-Covid-19 related ARDS (NC-ARDS) has been evaluated to be 22% [95% confidence interval (CI) 19–25%] during the first 72 h of protective mechanical ventilation [1]. A clinical risk score has been proposed to select NC-ARDS patients at risk of ACP, including four variables: pneumonia as a cause of ARDS, elevated driving pressure, severe hypoxemia and severe hypercapnia [1]. RV dysfunction has been also reported in the setting of COVID-19-related ARDS (C-ARDS) [2], but the prevalence of ACP and the validity of ACP risk score in C-ARDS patients are still unknown. We performed an observational study in the medical ICU of Henri Mondor University Hospital (Créteil, France), from March 9th 2020 to March 9th 2021 to assess the prevalence and predictors of ACP in C-ARDS.

Continuous data are expressed as the mean ± standard deviation or median [25th–75th percentiles] and were compared using the Student t test or Mann–Whitney U test, as appropriate. Categorical variables, expressed as number and percentages, were evaluated using the chi-square test or Fisher’s exact test. To evaluate independent factors associated with ACP, significant or marginally significant (*p* < 0.10) bivariate risk factors (using the above-mentioned tests) were examined using univariate and multivariable backward stepwise logistic regression analysis. Coefficients were computed by the method of maximum likelihood. The calibrations of model was assessed by the Hosmer–Lemeshow goodness-of-fit statistic and discrimination by the area under the receiver operating characteristics curve.

Among 282 Covid-19 patients admitted in our ICU during the study period, 175 were intubated and ventilated for C-ARDS. Fifty-eight C-ARDS patients were excluded because they had no available echocardiographic data obtained within 72 h of initiation of invasive mechanical ventilation and the remaining 117 patients were included. In our cohort, the observed prevalence of ACP (44/117, 38%, 95% confidence interval 0.29–0.47) was higher than previously described for NC-ARDS. C-ARDS patients with ACP were less likely to have diabetes or chronic kidney disease (Table 1). They were not more likely to have a thorax computed tomography angiogram performed but, if they did have the exam, they were significantly more likely to present a pulmonary embolism (Table 1). On the contrary, there was no significant association between the presence of ACP and the ACP risk score or its components (Table 1). In multivariable analysis, pulmonary embolism was the only factor associated with ACP (Table 2). Including the ACP risk score in the model yielded similar results. Patients with ACP had a trend towards more extracorporeal membrane oxygenation and required tracheostomy more frequently, but had a similar mortality than their counterparts (Table 1).

**Table 1:** Characteristics and outcomes of patients with Covid-19 related acute respiratory distress syndrome, with or without acute cor pulmonale

	N patients with data	All patients (n = 117)	No ACP (n = 73)	ACP (n = 44)	*p* value
Patient characteristics					
Age (years)	117	62.0 ± 10.3	63.2 ± 9.9	60.2 ± 10.9	0.132
Male gender	117	94 (80%)	60 (82%)	34 (77%)	0.517
Body mass index (kg/m^2^)	113	29.06 ± 5.69	28.27 ± 5.69	30.34 ± 5.50	0.061
SAPS II	116	36 [28–46]	37 [30–47]	34 [27–46]	0.249
SOFA score (Day 1)	117	5 [4–8.5]	5 [4–9]	5 [4–8]	0.503
Medical history					
Diabetes	117	47 (40%)	36 (49%)	11 (25%)	0.009
Arterial Hypertension	117	69 (59%)	47 (64%)	22 (50%)	0.125
Heart failure (NYHA III-IV)	117	9 (8%)	8 (11%)	1 (2%)	0.150
Chronic kidney disease	117	19 (16%)	17 (23%)	2 (5%)	0.008
Chronic obstructive pulmonary disease	117	11 (9%)	7 (10%)	4 (9%)	0.929
Respiratory parameters*					
pH	112	7.36 [7.31–7.41]	7.36 [7.31–7.42]	7.38 [7.33–7.41]	0.366
PaCO_2_ (mmHg)	112	42 [38–47]	42 [38–47]	44 [39–48]	0.313
P/F ratio	116	132 [95–177]	135 [96–175]	129 [91–189]	0.869
PEEP (cmH_2_O)	110	11 [9–12]	11 [8.75–12]	11.5 [9–12]	0.869
Driving Pressure (cmH_2_O)	100	12 [10–15]	13 [11–15]	12 [10–14]	0.108
Tidal Volume (mL/kg)	86	6.0 [5.7–6.4]	6.1 [5.9–6.4]	5.9 [5.6–6.5]	0.37
Respiratory Rate (/min)	83	30 [26–32]	28 [25–32]	30 [28–34]	0.126
Respiratory-system compliance (mL/cmH2O)	102	35 [28–40]	34 [27–40]	37 [29–44]	0.089
ARDS ACP risk score					
Pneumonia as cause of ARDS	117	117 (100%)	73 (100%)	44 (100%)	> 0.99
Driving pressure ≥ 18 cmH2O	100	11 (9%)	9 (12%)	2 (5%)	0.192
P/F < 150	116	77 (66%)	46 (63%)	31 (70%)	0.468
PaCO_2_ ≥ 48 mmHg	112	27 (23%)	16 (22%)	11 (25%)	0.773
Total ACP risk score (0–4)	97	2 [1–2.5]	2 [1, 2]	2 [1–3]	0.978
Laboratory data**					
Platelets (10^9^/L)	115	244 [182–303]	244 [182–298]	243 [189–312]	0.92
Fibrinogen (g/L)	94	6.82 ± 1.72	6.95 ± 1.65	6.62 ± 1.83	0.379
D-dimer (ng/mL)	84	1948 [1140–4205]	1948 [1249–2956]	2335 [1006–8660]	0.551
CT-scan data					
Thorax CT angiography***	117	81 (69%)	54 (74%)	27 (61%)	0.431
Pulmonary embolism	81	9 (8%)	2 (3%)	7 (16%)	0.007
ICU and outcome data****					
Prone position	117	107 (91%)	64 (88%)	43 (98%)	0.087
Shock	117	91 (78%)	55 (75%)	36 (82%)	0.414
Nitrous oxide use	117	26 (22%)	14 (19%)	12 (27%)	0.308
Tracheotomy	117	19 (16%)	8 (11%)	11 (25%)	0.046
VV-ECMO	117	22 (19%)	10 (14%)	12 (27%)	0.069
Ventilation days (survivors)	71	17 [10–38]	15 [8–34]	22 [11–42]	0.395
Ventilator-free days at D28	115	0 [0–13]	0 [0–15]	0 [0–13]	0.516
D28 all-cause mortality	117	44 (38%)	29 (40%)	15 (34%)	0.542

ACP: Acute *cor pulmonale*; CT: Computed Tomography Scan; NYHA: New York Heart Association; PEEP: Positive End-Expiratory Pressure; SAPS II: Simplified Acute Physiology Score II; SOFA: Sequential Organ Failure Assessment; VV-ECMO: Veno-Venous ExtraCorporeal Membrane Oxygenation

*Data obtained at the time of echocardiographic evaluation; **Data obtained within 48 h (either before or after) of the echocardiographic evaluation; ***CT scan was performed a median of 2 [0–4] days before the echocardiographic evaluation; **** Data regarding the totality of ICU stay

**Table 2:** Univariate and multivariable analysis for acute cor pulmonale in patients with Cocid-19 related acute respiratory distress syndrome

	N patients with data	Univariate analysis	Multivariable analysis
Patient characteristics*			
Body mass index (kg/m^2^)	113	1.07 [0.996–1.14], *p* = 0.06	NR
Medical history*			
Diabetes	117	0.34 [0.15–0.78], *p* = 0.01	NR
Chronic kidney disease	117	0.16 [0.03–0.71], *p* = 0.02	NS
Respiratory parameters*			
Respiratory system compliance (mL/cmH_**2**_O)	102	1.02 [0.99 -1.05], *p* = 0.27	NS
CT-scan			
Pulmonary embolism	81	8.5 [1.63–44.33], *p* = 0.01	7.30 [1.32–40.29], *p* = 0.02

Data presented as OR [95% CI]; OR—Odds Ratio, 95% CI—95% Confidence Interval, NS—not significant in the final model, NR—not retained in the final model; The multivariable model showed a good calibration as assessed by the Hosmer and Lemeshow goodness of fit test [*X*^2^(8 *df*) = 7.8, *p* = 0.45] and a fair discrimination as assessed by the receiver operating characteristics curve [area under the curve (AUC) 0.779; 95% CI 0.673–0.885; *p* < 0.0001]

Our study suggests that ACP is more prevalent in C-ARDS than previously reported in NC-ARDS, and is rather driven by pulmonary vascular obstruction in this group of patients than classical risk factors favoring vascular constriction/compression (hypoxemia, hypercapnia and driving pressure). Widespread pulmonary thrombosis with microangiopathy is a characteristic histological feature of C-ARDS [3, 4]. Pulmonary embolism is reported in up to 24% of critically-ill patients with C-ARDS [5]. Our data suggests that the presence of ACP may prompt the search of pulmonary embolism by a CT-scan in C-ARDS patients.

In conclusion, ACP seems more frequent and more related to pulmonary embolism in C-ARDS as compared to NC-ARDS. These observations need to be confirmed in larger studies.

## Data Availability

The dataset used during the current study is available from the corresponding author upon reasonable request.
